# Optimal Catheter Ablation Strategy for Patients with Persistent Atrial Fibrillation and Heart Failure: A Retrospective Study

**DOI:** 10.1155/2022/3002391

**Published:** 2022-06-23

**Authors:** Cheng-ming Ma, Ye-jian He, Wen-wen Li, Hua-min Tang, Shi-yu Dai, Xiao-meng Yin, Xian-jie Xiao, Yun-long Xia, Lian-jun Gao, Yuan-jun Sun, Zhong-zhen Wang, Rong-feng Zhang

**Affiliations:** ^1^Department of Cardiology, Institute of Cardiovascular Diseases, First Affiliated Hospital of Dalian Medical University, Dalian, China; ^2^Department of Cardiology, Central Hospital of Zhuanghe City, Dalian, China; ^3^Department of Critically Care Medicine, First Affiliated Hospital of Dalian Medical University, Dalian, China; ^4^Department of Graduate School, Dalian Medical University, Dalian, China

## Abstract

The optimal catheter ablation (CA) strategy for patients with persistent atrial fibrillation (PeAF) and heart failure (HF) remains uncertain. Between 2016 and 2020, 118 consecutive patients with PeAF and HF who underwent the CA procedure in two centers were retrospectively evaluated and divided into the pulmonary vein isolation (PVI)-only and PVI + additional ablation groups. Transthoracic echocardiography (TTE) was performed at baseline, one month, and 12 months after the CA procedure. The HF symptoms and left ventricular ejection fraction (LVEF) improvements were analyzed. Fifty-six patients underwent PVI only, and 62 patients received PVI with additional ablation. Compared with the baseline, a significant improvement in the LVEF and left atrial diameter postablation was observed in all patients. No significant HF improvement was detected in the PVI + additional ablation group than in the PVI-only group (74.2% vs. 71.4%, *P* = 0.736), but the procedure and ablation time were significantly longer (137.4 ± 7.5 vs. 123.1 ± 11.5 min, *P* = 0.001). There was no significant difference in the change in TTE parameters and the number of rehospitalizations. For patients with PeAF and HF, CA appears to improve left ventricular function. Additional ablation does not improve outcomes and has a significantly longer procedure time. Trial registration number is as follows: ChiCTR2100053745 (Chinese Clinical Trial Registry; https://www.chictr.org.cn/index.aspx).

## 1. Introduction

Heart failure (HF) remains a significant cause of mortality worldwide. Atrial fibrillation (AF) is a common type of arrhythmia in patients with HF [1]. AF and HF often facilitate the occurrence and exacerbate the outcome of each other as follows: AF increases the stroke risk, rehospitalization rate, morbidity, and mortality of HF; moreover, HF is a thromboembolic risk factor in AF [2]. Rhythm control, particularly catheter ablation (CA), has been shown to reduce AF-related symptoms and improve quality of life (QoL) compared with rate control [3, 4]. Several randomized clinical trials (RCTs) and meta-analyses comparing CA with pharmacological therapies in AF and HF have demonstrated that CA is a well-established treatment for improving QoL, HF hospitalization, and outcome [5–8]. Based on these RCTs, in their new guidelines, the American Heart Association (AHA) recommends CA as first-line therapy for patients with AF and HF with reduced ejection fraction (HFrEF) [9].

For paroxysmal AF (PaAF), pulmonary vein isolation (PVI) has always been the cornerstone of CA, with a satisfactory success rate and reliable safety, since recognising the crucial triggering role of the pulmonary veins [10]. However, the optimal CA strategy for PeAF is controversial, and the effectiveness of adjunctive linear ablation strategies beyond PVI is debated [11]. Whether additional atrial linear ablation improves the outcome of patients with PeAF and HF is unknown. Moreover, the low tolerance of HF patients may limit the duration of the CA procedure. To date, limited studies focusing on optimal CA strategies in patients with PeAF and HF have been reported. We retrospectively analyzed the impact of different ablation strategies on patients with PeAF and HF. This study aimed to clarify whether additional linear atrial ablation can reduce the number of rehospitalizations and improve outcomes, and to determine the optimal CA strategy for PeAF and HF patients.

## 2. Methods

### 2.1. Patients

This was a multicenter, retrospective study of 118 consecutive patients with PeAF and HF who underwent their first CA procedure in the First Affiliated Hospital of Dalian Medical University and the Central Hospital of Zhuanghe City between January 2016 and December 2020. Patients with previous ablation procedures for AF, congenital heart diseases, or cardiac surgical atriotomy were excluded. Written informed consent was obtained from all enrolled patients. The local ethics committees approved this study.

**Figure 1:**
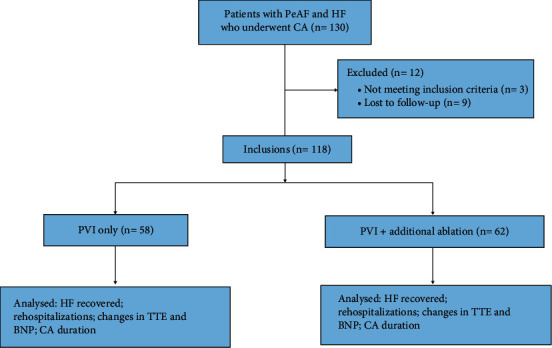
Flowchart of the included and analyzed patients. Of 118 patients with PeAF and HF enrolled, 58 patients received PVI alone and 62 patients received PVI and additional ablation.

According to the CA ablation strategy, the 118 patients were classified into a PVI-only group (Group A: *n* = 56) and a PVI plus additional ablation group (Group B: *n* = 62) (Figure 1).

### 2.2. CA Procedure

All patients received optimal HF management and at least three weeks of anticoagulation prior to the CA procedure. Antiarrhythmic drugs were discontinued for at least five half-lives. The presence of an atrial thrombus was excluded by transesophageal echocardiography (TEE) one day before or on the day of the procedure. During the CA procedure, a decapolar catheter was positioned in the coronary sinus for atrial pacing via the right femoral vein. Transseptal puncture guided by a modified Brockenbrough technique was carried out using 8 F long sheaths (SL1, Synaptic Medical, China). An initial heparin bolus was given immediately following the transeptal puncture, and the target ACT was maintained at 300–350 seconds by continuous heparin infusion. A PV mapping catheter (LASSO or PentaRay, Biosense Webster, Irvine, USA) was used for mapping PV potential. A saline-irrigated ablation catheter (THERMOCOOL SMART TOUCH SF, Biosense Webster, CA, USA or CoolFlex, EnSite-NavX, St. Jude Medical, MN, USA) was applied for mapping and ablation using electroanatomic mapping systems (CARTO3, Biosense Webster, CA, USA or EnSite-NavX, St Jude Medical, MN, USA).

The endpoint of CA for the PVI-only group was complete electrical PVI, as demonstrated by the absence of PV potentials or PV-left atrium conduction. A direct current electric conversion was performed, and then PVI was verified. In the PVI plus additional ablation group, additional ablation (left atrial roof, mitral isthmus, BOX, or complex fractionated atrial electrograms (CFAEs)) was performed to achieve a bidirectional block or to restore sinus rhythm. If the sinus rhythm was not restored, direct current electric conversion was carried out. If typical atrial flutter was documented before the procedure or induced during the procedure, cavotricuspid isthmus (CTI) ablation was performed.

### 2.3. Follow-Up

All patients were followed up monthly in the clinic for at least 1-year postablation. Anticoagulation therapy was prescribed for at least two months. All patients underwent standardized 2-dimensional transthoracic echocardiography (TTE) imaging at baseline, one month, and 12 months. The LVEF was determined according to Simpson's rule from left ventricular end-diastolic and end-systolic volumes in apical 5- and 2-chamber views. The parameters on TTE at postablation were calculated as the average of parameters at one month and 12 months. Successful CA was defined as no atrial tachycardia more than 30 s after the blanking period (3 months after the CA procedure) without antiarrhythmic drugs. The number of all-cause deaths and hospitalizations was obtained at 12 months. Thromboembolic and bleeding complications were also recorded.

The definition of HF with recovered LVEF (HFrecEF) was consistent with the majority of studies in the literature, including the following: (1) the documentation of a decreased LVEF <40% at baseline, (2) >10% absolute improvement in the LVEF, and (3) a second measurement of the LVEF >40% [12].

### 2.4. Statistical Analysis

Data analysis was performed with SPSS 22.0 (SPSS Inc., Chicago, USA). Quantitative data are expressed as the mean ± standard deviation (SD) if normally distributed and as the median [first quartile, third quartile] if nonnormally distributed. The homogeneity of variance was tested by the Kolmogorov–Smirnov Goodness-of-Fit test. Categorical data are expressed as frequencies and percentages. An unpaired *t*-test or nonparametric Mann–Whitney *U* test was performed for comparisons between groups for quantitative data. For categorical variables, chi-squared tests or Fisher's exact tests were used. A 2-tailed *P* value <0.05 was considered statistically significant.

## 3. Results

### 3.1. Patient Characteristics

A total of 118 patients (63.7 ± 10.1 years; 66.9% male; 57.6% HFrEF, 5.1% HFpEF, and 37.3% HFmrEF) were included in this study. Among them, 56 (47.5%) patients underwent PVI-only ablation (Group A), and 62 (52.5%) patients received additional ablation (Group B). The baseline characteristics of these patients are shown in Table 1. No significant differences in sex, age, comorbidity, AF duration, or New York Heart Association (NYHA) function classification were detected between the two groups.

### 3.2. CA Strategy

For all patients, the procedure time was 130.6 ± 12.0 min, and the ablation time was 94.9 ± 12.8 min. The procedure and ablation time in the PVI + additional ablation group were significantly longer than those in the PVI-only group (procedure time: 137.4 ± 7.5 min vs. 123.1 ± 11.5 min, *P*=0.001; ablation time: 104.5 ± 7.8 min vs. 84.2 ± 7.9 min, *P*=0.001, Table 1).

In the PVI + additional ablation group, 33 of 62 (53.23%) patients received additional linear ablation (including left atrial roof, mitral isthmus, posterior wall, or BOX isolation), 23 (37.1%) patients received CTI, and 3 (4.84%) patients received linear ablation + CFAE ablation (Additional Figure 1).

### 3.3. HFrecEF

After a mean follow-up period of 34.2 ± 14.7 months, a significant improvement in LVEF postablation was observed in all patients. The mean LVEF rose from a baseline mean of 38.4% (38.4 ± 8.7%) to a postablation mean of 49.2% (49.2 ± 9.8%) (Additional Figure 2). These EFs of 86 of 118 patients recovered; however, no significant difference was detected between the PVI-only group and the PVI + additional ablation group (71.4% (40 of 56) vs. 74.2% (46 of 62), *P*=0.736) (Table 2).

### 3.4. Number of Rehospitalizations and Changes in TTE Parameters and BNP

**Figure 2:**
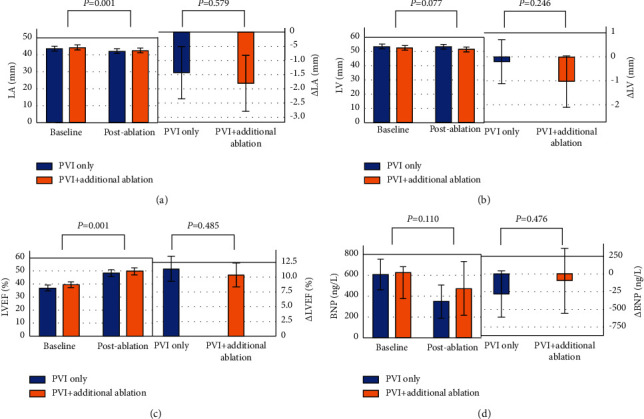
Clustered bar chart of secondary endpoints at baseline and postablation and of the change from baseline to postablation.

Compared with the baseline, a significant improvement in the LVEF and left atrial diameter postablation was observed in all patients (Table 3). However, no significant difference in the changes was found between the two groups (Table 2 and Figure 2). Additionally, there was no significant difference in the number of rehospitalizations between the two groups (1.2 ± 1.8 vs. 0.94 ± 1.5, *P*=0.27).

## 4. Discussion

In the present study, we found that CA of AF for patients with HF and PeAF can significantly improve the LVEF and decrease the LA diameter. Compared with PVI only, additional ablation beyond PVI did not significantly improve the outcome of patients with HF and had a longer procedure time. Thus, PVI alone may be more appropriate for patients with HF and PeAF.

Several RCTs have shown that rhythm control, especially CA, is superior to rate control for patients with HF and AF [2, 13]. The CAMERA-MRI study (Catheter Ablation versus Medical Rate Control in Atrial Fibrillation and Systolic Dysfunction) revealed that the restoration of sinus rhythm with CA results in more significant improvements in left ventricular function [14]. These improvements were also observed in the atrial structure and the long-term follow-up [15]. In the CASTLE-AF (Catheter Ablation vs. Standard Conventional Treatment in Patients with LV Dysfunction and AF) trial of patients with HFrEF and AF, compared with medical therapy, a significantly lower rate of death or worsening HF was detected with CA [6]. The AATAC (Ablation vs. Amiodarone for Treatment of Atrial Fibrillation in Patients With Congestive Heart Failure and an Implanted ICD/CRTD) trial demonstrated that CA is superior to amiodarone in reducing AF recurrence, the number of hospitalizations, and mortality in patients with PeAF and HFrEF [8]. This improvement was more pronounced in recurrence-free patients than in the recurrence group. A meta-analysis of 11 RCTs comparing antiarrhythmic drug (AAD) rhythm control vs. rate control or CA rhythm control vs. medical therapy in patients with AF and HF demonstrated that CA improves the survival rate, rehospitalization rate, maintenance of sinus rhythm, and cardiac function [16]. Based on these RCTs, in the recent guidelines of the AHA, they suggested that CA may be considered the first-line treatment in patients with AF and HFrEF [9]. In our study, there was a significant increase in the number of patients with HF and PeAF who underwent the CA procedure in the last three years, demonstrating an increased recognition of and confidence in CA for these patients. The effectiveness of CA in patients with seriously advanced HF could be limited. The AMICA (Atrial Fibrillation Management in Congestive Heart Failure With Ablation) trial, comparing CA and the best medical therapy in patients with PeAF and HFrEF (LVEF <35%), did not find a better clinical benefit in CA [7]

The effect of CA on AF in patients with HF with preserved EF (HFpEF) is unclear. The restoration of sinus rhythm is critical for improving outcomes in patients with HFpEF [17]. A subgroup analysis of 778 patients of the CABANA trial (Catheter Ablation Versus Anti-arrhythmic Drug Therapy for Atrial Fibrillation) demonstrated that CA significantly improved survival, QoL, and freedom from AF [18]. Almost all patients with HF enrolled had a preserved EF; therefore, it is reasonable to expect that CA can also improve the outcome of HFpEF. In our study, only 14.4% of all the enrolled patients had an LVEF <30%, and 5.5% of patients had HFpEF, indicating that these patients rarely received the CA procedure or did not progress into HF. Recently, the EAST-AFNET 4 trial (Early Treatment of Atrial Fibrillation for Stroke Prevention Trial) revealed that early rhythm control of AF could decrease the risk of developing conditions in patients with HF [19]. This clinical benefit was observed in all HF patients, regardless of the baseline LVEF [20].

The results and outcome of CA in patients with PeAF are currently unsatisfactory. It is well known that arrhythmogenic mechanisms beyond the pulmonary veins are often involved in patients with PeAF. The prevailing CA strategies beyond PVI for PeAF include targeting mechanisms and anatomical ablation, and recently, Zefferino Palam et al. have well summarized the current CA strategies in this article [21]. CFAEs and rotor ablation have been developed in the past ten years to eliminate PeAF, but did not demonstrate more benefits than PVI alone. Substrate definition and identification, and recognize patients who may benefit from the emerging techniques need further study to improve the CA results. Low-voltage area identification by high-density endocardial voltage mapping and fibrosis identification using cardiac magnetic resonance imaging may be effective approaches [22, 23]. Some non-PV triggers have been proven to be involved in AF, including left atrial appendage (LAA), posterior wall, superior vena cava, coronary sinus, and CTI. Some adjunctive ablation strategies have been evaluated to improve CA outcomes for patients with PeAF. Unfortunately, the success rate is disappointing, and early studies and meta-analyses did not find a reduction in AF recurrence when additional ablation was performed [11, 24, 25]. Moreover, the extensive ablation strategy may result in new arrhythmogenic foci. Achieving a durable bidirectional block and avoiding incomplete ablation are crucial issues for linear ablation. A meta-analysis involving nine studies evaluating the effect of LAA isolation in nonparoxysmal AF reported that LAA isolation is associated with a significant long-term improvement in freedom from all-atrial arrhythmia recurrence [26]. However, LAA isolation is challenging; it requires a long procedural time with an experienced operator, the reconnection rate may be high, and the risk of LAA thrombus is potentially increased [27]. The efficacy of posterior wall isolation (PWI) in PeAF, such as box isolation, has been established with inconsistent findings in relevant RCTs. In a meta-analysis of 17 studies, PWI seemed easy to achieve with a relatively satisfactory single-procedure success rate but was not competitive with PVI [28]. The vein of Marshall ethanol infusion with CA may be another choice for PeAF [29]. In our study, over half of the PVI + additional ablation patients received linear ablation, including left atrial roof, mitral isthmus, and BOX. Currently, there is no standard CA strategy for PeAF, and a reasoned approach guided by high-density mapping or other new mapping technologies may make a tailored ablation strategy possible.

It seems that PVI alone is not sufficient for PeAF, but relevant clinical trials have demonstrated that PVI alone can serve as a sole strategy for PeAF. In the PRECEPT (persistent atrial fibrillation ablation with contact force sensing catheter) trial, including patients with PeAF <1 year, the primary ablation procedure was PVI-targeted with an overall clinical success rate of 80%, although 45% of patients received additional ablation [30]. These results were better than those in the STAR AF II (substrate and trigger ablation for reduction of atrial fibrillation trial-Part II) trial, in which patients with PeAF for up to three years were enrolled, and the findings supported that early rhythm control is essential to PeAF. The CRYO4PERSISTENT AF trial, assessing the outcome of PVI using only a cryoballoon in patients with PeAF, achieved a satisfactory success rate [31]. A similar result was observed in a multicenter study of PeAF or longstanding PeAF, indicating that PVI alone is a reliable ablation strategy for PeAF [32]. To date, most studies have suggested that the effect of additional ablation is negative, thus, in the current clinical practice, an individualized ablation strategy is often adopted for patients with PeAF. [33]. An individualized CA strategy for PeAF may be more crucial in the context of HF. In the present study, we did not find any further improvement in HF in the PVI + additional ablation group; in contrast, the procedure time was significantly longer than that for PVI only. Considering the low tolerance of patients with HF, a short procedure time is more appropriate and safer.

This study has some limitations. First, this is a retrospective study. However, most patients underwent the CA procedure within the last three years, and the procedures were performed by four experienced operators, and only 7.1% of patients were lost to follow-up, helping to minimize bias. Second, the sample size was limited, but there were relatively few patients with HF and PeAF who were willing to undergo CA. Third, ECG and 24-h Holter monitoring in the clinic, rather than an insertable cardiac monitor, have the potential to underestimate AF recurrence. Not all patients underwent 24 h Holter monitoring; thus, we cannot accurately measure AF recurrence. Fourth, this study did not mention some CA strategies, such as ablation of intramural ganglionated plexuses, Marshall ligament, and rotors, as they were not carried out in our center. Finally, a multipolar catheter that allows a better characterization of the atrial substrate, such as PentaRay, was not used in all patients in our study.

## 5. Conclusions

For patients with PeAF and HF, CA appears to improve the left ventricular function. Additional ablation beyond PVI is common in CA of PeAF; however, it does not improve outcomes and had a significantly longer procedure time. Therefore, PVI alone may be more appropriate for patients with HF and PeAF.

## Figures and Tables

**Table 1:** Baseline clinical characteristics of the patients.

	Total (*n* = 118)	PVI only (*n* = 56)	PVI + additional ablation (*n* = 62)	*P*
Age (years)	63.7 ± 10.1	63.2 ± 9.9	64.1 ± 10.3	0.641
Male	79 (66.9%)	42 (75%)	37 (59.7%)	0.077
CAD	48 (40.7%)	25 (44.6%)	23 (37.1%)	0.405
Hypertension	49 (41.5%)	28 (50%)	21 (33.9%)	0.076
Diabetes mellitus	25 (21.2%)	13 (23.2%)	12 (19.4%)	0.608
Stroke/TIAs	12 (10.2%)	5 (8.9%)	7 (11.3%)	0.672
NYHA classification				0.493
I	3 (2.5%)	1 (1.8%)	2 (3.2%)	—
II	41 (34.7%)	22 (39.3%)	19 (30.6%)	—
III	65 (55.1%)	29 (51.8%)	36 (58.1%)	—
IV	9 (7.6%)	4 (7.1%)	5 (8.1%)	—
CHA_2_DS_2_-VASc score	2.4 ± 1.5	2.3 ± 1.4	2.5 ± 1.6	0.429
LAD (mm)	44.0 ± 4.9	43.7 ± 5.4	44.3 ± 4.5	0.519
LVD (mm)	53.2 ± 6.2	53.8 ± 6.3	52.8 ± 6.1	0.426
LVEF (%)	38.4 ± 8.7	37.1 ± 8.2	39.6 ± 9	0.117
AF duration (years)	2.7 ± 1.8	2.6 ± 1.6	2.9 ± 1.9	0.370
Average HR (BPM)	89.4 ± 17.11	88.8 ± 14.9	89.9 ± 18.8	0.768
BNP (pg/mL)	465.3 [214.2, 745.6]	431.4 [245.4, 821.7]	376.9 [153.9, 738.8]	0.455
Creatinine (*μ* mol/L)	82.3 ± 20.6	84.3 ± 20.7	80.1 ± 20.4	0.318
ALT	38.3 ± 27.9	38.8 ± 25.2	37.6 ± 31.0	0.836
AST	30.6 ± 19.2	29.7 ± 15.7	31.5 ± 22.6	0.670
Hemoglobin (g/L)	148.3 ± 17.4	150.4 ± 16.1	146.1 ± 18.6	0.224
HDL (mmol/L)	2.2 ± 7.9	2.1 ± 7.5	2.4 ± 8.4	0.862
LDL (mmol/L)	4.9 ± 17.3	4.5 ± 16.1	5.3 ± 18.6	0.838
TC (mmol/L)	9.1 ± 32.5	8.6 ± 31.0	9.6 ± 33.1	0.892
Procedure time (min)	130.6 ± 12.0	123.1 ± 11.5	137.4 ± 7.5	0.001
Ablation time (min)	94.9 ± 12.8	84.2 ± 7.9	104.5 ± 7.8	0.001

BPM: beats per minute; CAD: coronary artery disease; TIA: transient ischemic attacks; LAD: left atrial diameter; LVD: left ventricular diameter; LVEF: left ventricular ejection fraction; NYHA: New York Heart Association; BNP: brain natriuretic peptide; HDL: high-density lipoprotein; LDL: low-density lipoprotein; TC: total cholesterol.

**Table 2:** Clinical outcomes of the patients and changes in TTE parameters and BNP.

	PVI only (*n* = 56)	PVI + additional ablation (*n* = 62)	*P*
Recovered EF (*n*)	40 (71.4%)	46 (74.2%)	0.736
Number of rehospitalizations	1.2 ± 1.8	0.94 ± 1.5	0.270
∆ LA	−1.4 ± 3.4	−1.8 ± 3.8	0.579
∆ LVD	−0.2 ± 3.3	−1.0 ± 4.1	0.246
∆ LVEF	11.3 ± 7.9	10.3 ± 7.8	0.485
∆ BNP (ng/L)	−280.7 ± 755.6	−93.3 ± 213.5	0.476

**Table 3:** Baseline vs. postablation.

	Baseline (*n* = 118)	Postablation (*n* = 118)	*P*
LA (mm)	44.0 ± 4.9	42.4 ± 5.1	0.001
LVD (mm)	53.3 ± 6.2	52.6 ± 5.6	0.077
LVEF (%)	38.4 ± 8.7	49.2 ± 9.8	0.001
BNP (ng/L)	465.3 [214.2, 745.6]	189.5 [79.3, 497.1]	0.110

## Data Availability

The data generated or analysed during this study are included within the article.

## Supplementary Materials

Supplementary Materials Additional Figure 1 shows the components of additional ablation in the PVI + additional ablation group. Additional Figure 2 shows changes in LVEF after the CA procedure.
