# Supplementary figures and images for: Optimal Catheter Ablation Strategy for Patients with Persistent Atrial Fibrillation and Heart Failure: A Retrospective Study
Source: Cardiol Res Pract. 2022 Jun 23;2022:3002391. doi: 10.1155/2022/3002391 (PMC9246569; doi:10.1155/2022/3002391)

**Additional figure 1**


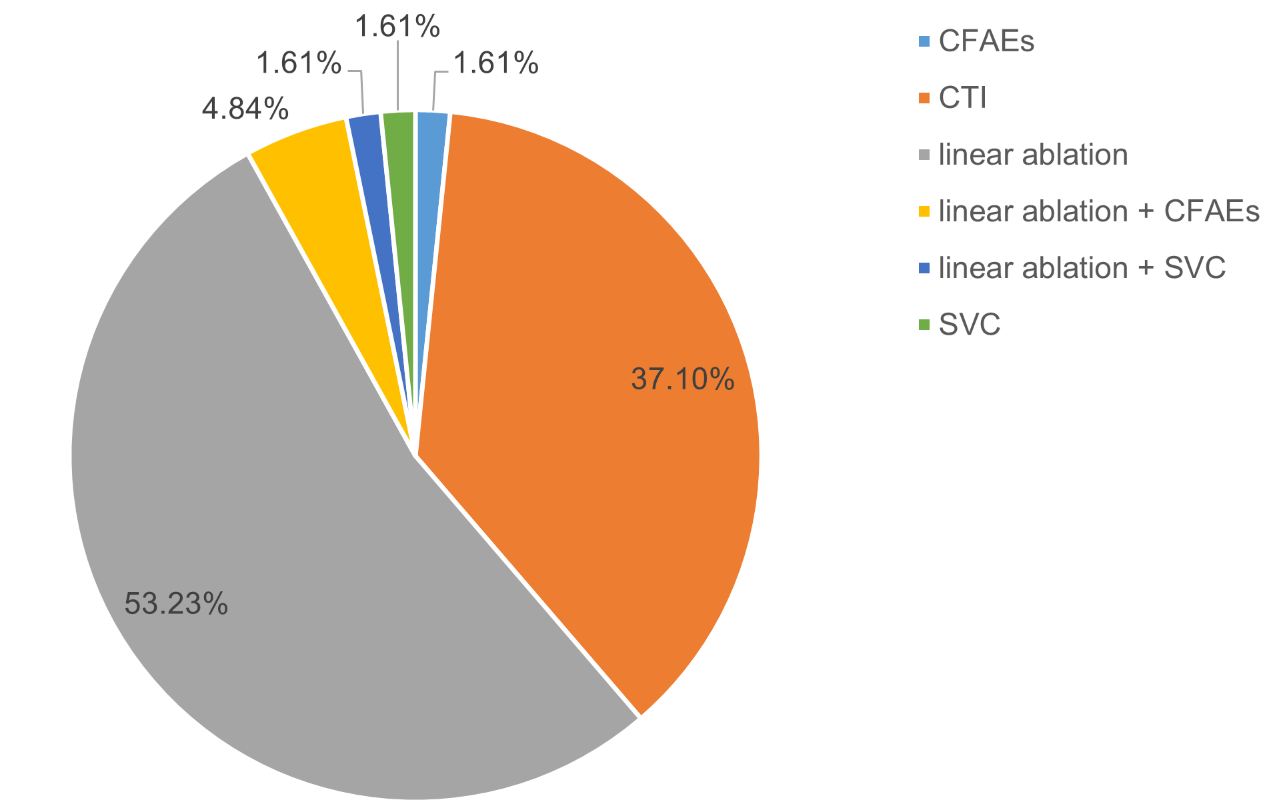


**Additional figure 2**


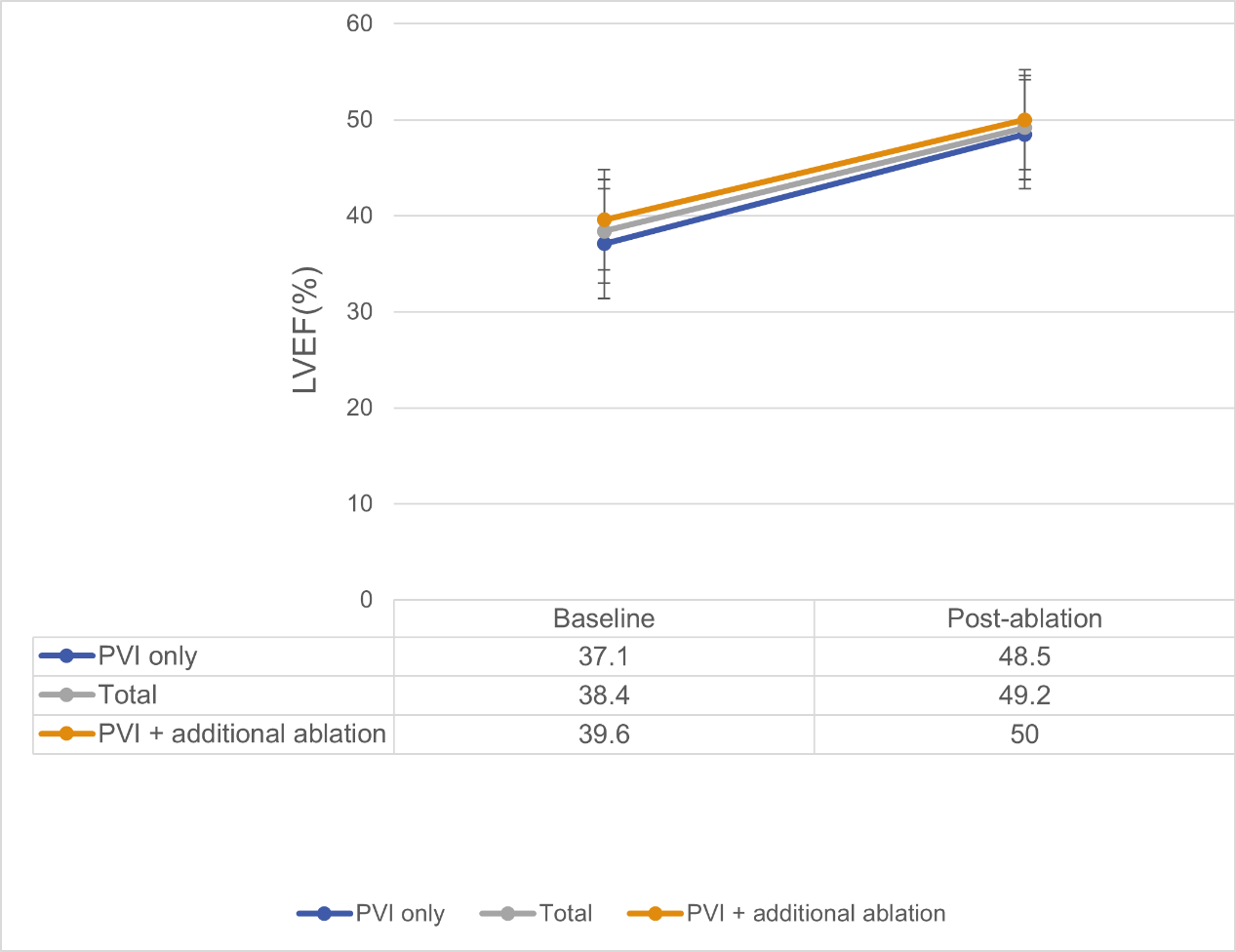

Supplement: Supplementary Materials — Additional Figure 1 shows the components of additional ablation in the PVI + additional ablation group. Additional Figure 2 shows changes in LVEF after the CA procedure. [file 3002391.f1.docx]
